# Post-acute COVID-19 syndrome in patients after 12 months from COVID-19 infection in Korea

**DOI:** 10.1186/s12879-022-07062-6

**Published:** 2022-01-27

**Authors:** Yoonjung Kim, Bitna-Ha, Shin-Woo Kim, Hyun-Ha Chang, Ki Tae Kwon, Sohyun Bae, Soyoon Hwang

**Affiliations:** Division of Infectious Disease, Department of Internal Medicine, Kyungpook National University Hospital, School of Medicine, Kyungpook National University, 130, Dongdeok-ro, Jung-gu, Daegu, 41944 Korea

**Keywords:** COVID-19, SARS-CoV-2, Sequelae, Persistent symptoms, Long COVID, Quality of life

## Abstract

**Background:**

As the coronavirus disease 2019 (COVID-19) pandemic continues to progress, awareness about its long-term impacts has been growing. To date, studies on the long-term course of symptoms, factors associated with persistent symptoms, and quality of life after 12 months since recovery from acute COVID-19 have been limited.

**Methods:**

A prospective online survey (First: September 8, 2020–September 10, 2020; Second: May 26, 2021–June 1, 2021) was conducted on recovered patients who were previously diagnosed with COVID-19 between February 13, 2020 and March 13, 2020 at Kyungpook National University Hospital. Responders aged between 17 and 70 years were included in the study. Overall, 900 and 241 responders were followed up at 6 and 12 months after recovery from COVID-19 in the first and second surveys, respectively. Clinical characteristics, self-reported persistent symptoms, and EuroQol-5-dimension (EQ5D) index score were investigated for evaluating quality of life.

**Results:**

The median period from the date of the first symptom onset or COVID-19 diagnosis to the time of the survey was 454 (interquartile range [IQR] 451–458) days. The median age of the responders was 37 (IQR 26.0–51.0) years, and 164 (68.0%) responders were women. Altogether, 11 (4.6%) responders were asymptomatic, and 194 (80.5%), 30 (12.4%), and 6 (2.5%) responders had mild, moderate, and severe illness, respectively. Overall, 127 (52.7%) responders still experienced COVID-19-related persistent symptoms and 12 (5.0%) were receiving outpatient treatment for such symptoms. The main symptoms were difficulty in concentration, cognitive dysfunction, amnesia, depression, fatigue, and anxiety. Considering the EQ5D index scores, only 59.3% of the responders did not have anxiety or depression. Older age, female sex, and disease severity were identified as risk factors for persistent neuropsychiatric symptoms.

**Conclusion:**

COVID-19-related persistent symptoms improved over time; however, neurological symptoms can last longer than other symptoms. Continuous careful observation of symptom improvement and multidisciplinary integrated research on recovered COVID-19 patients are required.

**Supplementary Information:**

The online version contains supplementary material available at 10.1186/s12879-022-07062-6.

## Background

To date, more than 200 million people have contracted coronavirus disease 2019 (COVID-19) globally. With the progression of COVID-19 pandemic, awareness about its long-term impacts has been growing steadily. According to previous studies, persistent symptoms after COVID-19 infection manifest in various forms. The most common symptoms are chronic fatigue, anosmia, ageusia, general neurological deterioration, and dyspnea [1–4]. At present, why long-term persistent symptoms are identified in recovered COVID-19 patients is unclear. However, most researchers and clinicians hypothesize that the long-term persistent symptoms after recovery from COVID-19 are associated with the ability of SARS-CoV-2 to trigger a massive inflammatory response [1]. Persistent symptoms are often associated with the severity of symptoms during the acute illness phase; however, long-lasting symptoms can also occur following mild illness and across all ages [5, 6]. Among the persistent symptoms, a key concern is the neurological complications of COVID-19 [7]. The impact of SARS-CoV-2 infection can have long-term negative impacts on cognitive functions, daily functioning, and quality of life [8–10]. A large proportion of patients experience persistent cognitive problems with memory loss and impaired ability to concentrate for several months after their recovery from COVID-19 [11]. Cognitive impairments were the most pronounced in people who had been hospitalized. Most importantly, cognitive impairments were also observed in non-hospitalized patients who were no longer reporting symptoms [12]. Until now, the risk factors associated with major neurological persistent symptoms that last more than 12 months are not yet known. Furthermore, to the best of our knowledge, studies on persistent symptoms and long-term consequences of COVID-19 on health-related quality of life and functional status after 12 months of recovery from an acute COVID-19 infection are limited [2, 3, 13]. Daegu was the first city where a severe COVID-19 outbreak affected more than 5000 individuals in early 2020 in Korea, thereby making it a suitable place for the investigation of the long-term COVID-19-related persistent symptoms. Therefore, we investigated the persistent symptoms in recovered patients in Daegu city to identify such symptoms prevalent 12 months after COVID-19 infection, factors associated with the major neurological persistent symptoms, and the long-term impact of COVID-19 on the quality of life in recovered COVID-19 patients.

## Methods

### Study design and population

This prospective online survey was conducted two times by infectious disease physicians at the Kyungpook National University Hospital located in Daegu city. The first survey period was from September 8, 2020 to September 10, 2020. In total, 5252 patients with COVID-19 (age 16–70 years) confirmed through polymerase chain reaction in Daegu city were identified from the clinical data registry provided by the Daegu Center for Infectious Diseases Control and Prevention and all included patients had in-hospital care or care in facility isolation centers. The date of diagnosis of the COVID-19 patients enrolled in the first survey was between February 18, 2020 and March 14, 2020. Among the 5252 patients in the first survey, 900 subjects were followed up for the initial 6 months after COVID-19 symptom onset or diagnosis. The first survey response rate was 17.1% (900 out of 5252). The second survey followed up after 12 months from acute COVID-19 infection was conducted from May 26, 2021 to June 1, 2021. A total of 7201 patients diagnosed with COVID-19 between February 17, 2020 and February 25, 2021 were identified from the clinical data registry provided by the Daegu Center for Infectious Diseases Control and Prevention. To distinguish and match responders in the first online survey with those in the second online survey, sex, birth year and date, and last four digit numbers of cellular phone were used.

The individualized questionnaire list included sex, birth year, birth date, residential address, COVID-19 diagnosis date, quarantine end date, symptoms identified during or after acute COVID-19 infection, oxygen treatment history, admission place during acute COVID-19, an open text field to add other persistent symptoms, newly diagnosed diseases after COVID-19, exacerbation of underlying diseases after COVID-19, and outpatient treatment history for COVID-19-related persistent symptoms or signs. Clinical data of the respondents in terms of COVID-19 diagnosis date, first symptom onset date, and disease severity were provided by the registry of Daegu Center for Infectious Diseases Control and Prevention. All data were reviewed by infectious disease physicians. EuroQol-5 dimension (EQ5D) index score was used to assess the quality of life associated with COVID-19-related persistent symptoms. EQ5D index score was classified into five categories as follows: Mobility, self-care, usual activities (e.g., work, study, housework, family, or leisure activities), Pain/Discomfort, and Anxiety/Depression.

All respondents in the study were diagnosed with SARS-CoV-2 infection via real-time reverse transcription polymerase chain reaction assay using nasopharyngeal swabs or other upper respiratory tract specimens. Respondents were considered recovered if they were no longer experiencing symptoms at the time of completion of the survey. End of quarantine period was defined as having two consecutive negative PCR results within a 24-h interval.

Severity scores during acute COVID-19 infection were classified as follows: (1) asymptomatic: no symptoms or discomfort throughout the entire disease period, with body temperature < 37.5 °C; (2) mild: presence of symptoms with or without fever (≥ 37.5 °C), but not manifesting or identified pneumonia; (3) moderate: pneumonia diagnosed by a clinician, but not requiring oxygenation other than room air; (4) severe: pneumonia diagnosed by a clinician, which requires oxygenation therapy (nasal prong, facial mask, or high-flow oxygen therapy); and (5) critical: pneumonia diagnosed by a clinician and need for mechanical ventilation therapy and/or extracorporeal membrane oxygenation or death [4].

### Assessment of symptoms

Clinical symptoms were classified into 45 symptoms and signs, which include fever, feeling feverish and feeling cold, myalgia, arthralgia, fatigue (a different decline in physical strength compared with the pre-disease state), cough, sore throat, rhinitis, sputum, dyspnea, palpitation, arrhythmia, chest discomfort, chest tightness, headache, dizziness, brain fog (the feeling of being mentally slow, fuzzy, or spaced out), cognitive dysfunction (loss of intellectual functions, such as thinking, remembering, and reasoning, severely enough to interfere with daily functioning), concentration difficulty, amnesia (loss of memory and inability to recall facts, information, and experiences), abnormal directional sensibility, insomnia, hyperemia, hypoacusis, tinnitus, hallucination, social phobia, seizure, depression, anxiety, posttraumatic stress disorder (PTSD) suspicious or diagnosed by physicians, obsessive thinking, anorexia, diarrhea, gastrointestinal discomfort, nausea or vomiting, anosmia, ageusia, paresthesia, alopecia, skin rashes, itchy skin, swollen fingers, and COVID toes (painful red or purple lesions that typically form on the fingers or toes).

### Statistical analysis

A descriptive analysis was conducted. Continuous variables were presented as median (interquartile range, IQR) values and categorical variables were presented as numbers (percentage, %). Categorical variables were analyzed using Fisher’s exact test or chi-square test and noncategorical variables were analyzed via Student’s *t*-test or Mann–Whitney U-test to compare the differences between respondents with and without persistent symptoms and for comparison of age, severity group, and sex differences according to the persistent symptoms. Multivariate regression analysis was performed to determine the factors associated with the major persistent symptoms. All the tests were considered statistically significant at *P* < 0.05. All statistical analysis was performed using R statistics version 4.0.2 (The R Foundation; https://www.r-project.org).

### Ethics statement

This study was reviewed and approved by the Institutional Review Board of Kyungpook National University Hospital (approval no.: 2021-02-003). All respondents provided digital informed consents before the questionnaire was administered. Without the informed consent, the remaining questionnaire could not be completed. All the study protocols were in accordance with relevant guidelines and regulations.

## Results

### Demographics and characteristics

The survey response rate was 9.4% (678 out of 7201 respondents). Of the total of 678 responders, 241 responders diagnosed with COVID-19 between February 18, 2020 and March 13, 2020 participated in the first online survey. The median days taken from the diagnosis of COVID-19 to the investigation period were 454 days [IQR 451.0; 458.0]. The total number of women was 164 (68.0%). The median age was 37.0 years [IQR 26.0; 51.0]. In addition to participants aged 60–70 years (n = 20, 8.3%), most of the participants (n = 99, 41.1%) were aged 17–29 years. At the time of acute COVID-19 infection, 194 (80.5%) responders were classified as mild, 30 (12.4%) as moderate, 11 (4.6%) as asymptomatic, and 6 (2.5%) as severe; however, none were classified as critical. Hospitalization sites during isolation were 132 (54.8%), therapeutic living centers (isolation facilities for asymptomatic COVID-19 patients) were 106 (44%), and self-isolation was (1.2%). Among those whose symptoms lasted up to 12 months, 98 (77.2%) were women; however, 66 (57.9%) women responded that they did not have symptoms that lasted up to 12 months (Table 1). No differences in sex distribution were identified in subgroup analyses between the participant aged < 50 years and those aged ≥ 50 years in the group with persistent symptoms (*P* = 0.342) (Additional file 1: Table S1). Those who responded had symptoms that persisted for more than 12 months and a higher median age of 41 years, as compared to the asymptomatic median age of 30 (*P* = 0.017); however, no statistically significant difference was identified in the age groups classified according to 10-year intervals (*P* = 0.181). Persistent symptoms for more than 12 months were more in those classified as moderate or higher disease severity during acute COVID-19 infection (*P* = 0.018) (Table 1). Seventy-nine (32.8%, 79/241) responders reported that they had a newly diagnosed disease due to COVID-19 infection. Twelve (5.0%, 12/241) responders were newly diagnosed with high blood pressure, 8 (3.3%, 8/241) responders were diagnosed with diabetes, and 4 (1.7%, 4/241) responders were newly diagnosed with liver disease.

**Table 1:** Clinical characteristics of 241 respondents according to the presence of persistent symptoms or signs identified after 12 months of recovery from acute COVID-19 infection

Characteristics	Total (N = 241)	No symptom (N = 114)	Symptom (N = 127)	*P* value
Days from COVID-19 diagnosis to survey, median [IQR]	454.0 [451.0–458.0]	453.0 [450.0–456.0]	455.0 [452.0–458.0]	0.009
Days from COVID-19-related symptom onset to diagnosis, median [IQR]	1.0 [0.0–4.0]	0.0 [0.0–3.0]	1.0 [0.0–5.0]	0.015
Sex				0.002
Male	77 (32.0%)	48 (42.1%)	29 (22.8%)	
Female	164 (68.0%)	66 (57.9%)	98 (77.2%)	
Age, median [IQR] (years)	37.0 [26.0–51.0]	30.0 [25.0–49.0]	41.0 [26.0–52.5]	0.017
Age distribution (years)				0.181
17–29	99 (41.1%)	55 (48.2%)	44 (34.6%)	
30–39	31 (12.9%)	15 (13.2%)	16 (12.6%)	
40–49	41 (17.0%)	17 (14.9%)	24 (18.9%)	
50–59	50 (20.7%)	21 (18.4%)	29 (22.8%)	
60–70	20 (8.3%)	6 (5.3%)	14 (11.0%)	
Age (years)				0.111
< 50	171 (71.0%)	87 (76.3%)	84 (66.1%)	
≥ 50	70 (29.0%)	27 (23.7%)	43 (33.9%)	
Disease severity				< 0.001
Asymptomatic	11 (4.6%)	11 (9.6%)	0 (0.0%)	
Mid	194 (80.5%)	93 (81.6%)	101 (79.5%)	
Moderate	30 (12.4%)	10 (8.8%)	20 (15.7%)	
Severe	6 (2.5%)	0 (0.0%)	6 (4.7%)	
Critical	0 (0.0%)	0 (0.0%)	0 (0.0%)	
Disease severity				
< Moderate	205 (85.1%)	104 (91.2%)	101 (79.5%)	0.018
≥ Moderate	36 (14.9%)	10 (8.8%)	26 (20.5%)	
Isolation period, median [IQR]	27.0 [21.0–37.0]	27.0 [20.0–36.0]	27.0 [22.0–38.0]	0.444
Isolation period				0.633
< 3 weeks	59 (24.5%)	30 (26.3%)	29 (22.8%)	
≥ 3 weeks	182 (75.5%)	84 (73.7%)	98 (77.2%)	
Isolated place*				
Secondary or Tertiary hospital	132 (54.8%)	49 (43.0%)	83 (65.4%)	0.002
Therapeutic living center	106 (44.0%)	63 (55.3%)	43 (33.9%)	
Self-home isolation	3 (1.2%)	2 (1.8%)	1 (0.8%)	
Underlying psychiatric disease				1.000
No	249 (99.2%)	113 (99.1%)	126 (99.2%)	
Yes	2 (0.8%)	1 (0.9%)^a^	1 (0.8%)^b^	

*Isolated place: The place where patients were quarantined after being diagnosed with COVID-19 for the first time

^a^Panic disorder

^b^Major depressive disorder, panic disorder

Eighty-three (34.4%, 83/241) responders reported the worsening of underlying diseases. Seven (2.9%, 7/241) responders reported that their high blood pressure worsened and 8 (3.3%, 8/241) responders reported that their diabetes worsened. Twelve (5.0%, 12/241) responders were undergoing outpatient treatment after 12 months from symptom onset or diagnosis. Among these 12 responders, 4 (5.7%, 4/70) were aged over 50 years and 8 (4.7%, 8/171) were aged under 50 years, which implied that the proportion of responders aged ≥ 50 years who were treated in outpatient clinics for COVID-19-related persistent symptoms was higher than those aged < 50 years.

### Characteristics of persistent symptoms and quality of life assessment

**Fig. 1:**
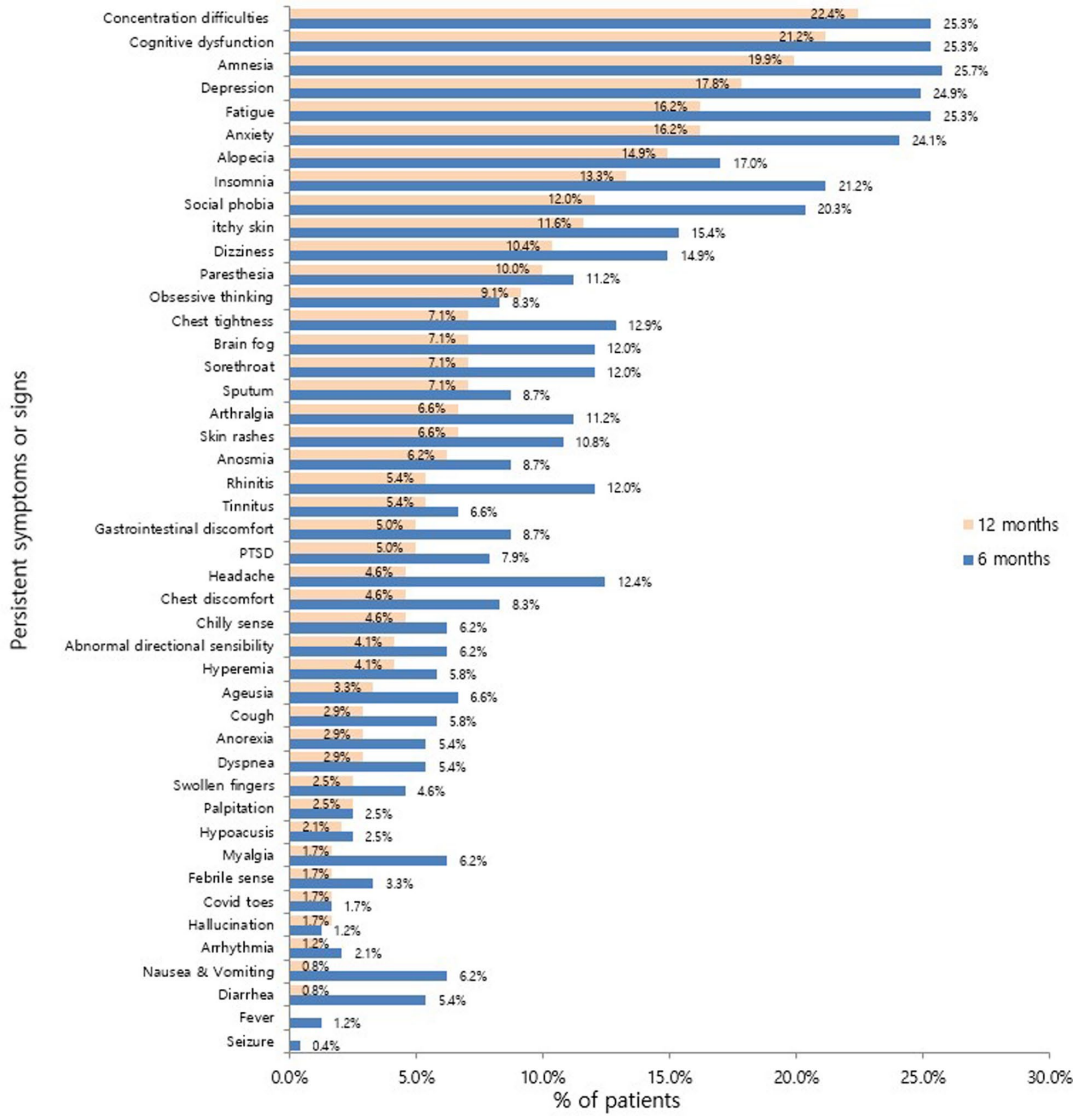
Distribution of 45 persistent symptoms or signs over 12 months after acute COVID-19 infection

**Fig. 2:**
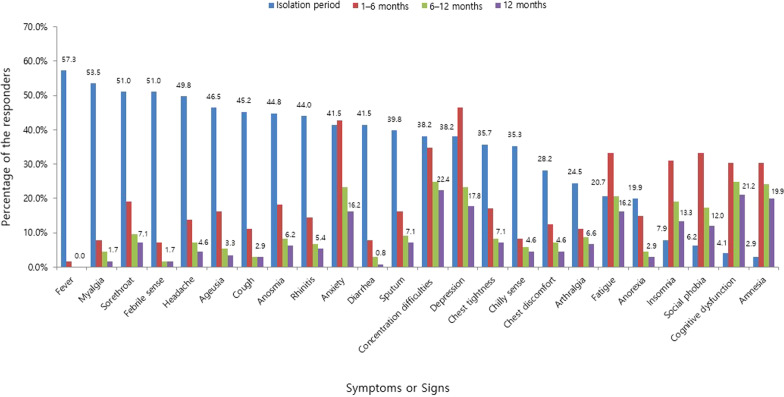
Persistent symptoms or signs according to the most frequent symptoms identified during isolation period

**Fig. 3:**
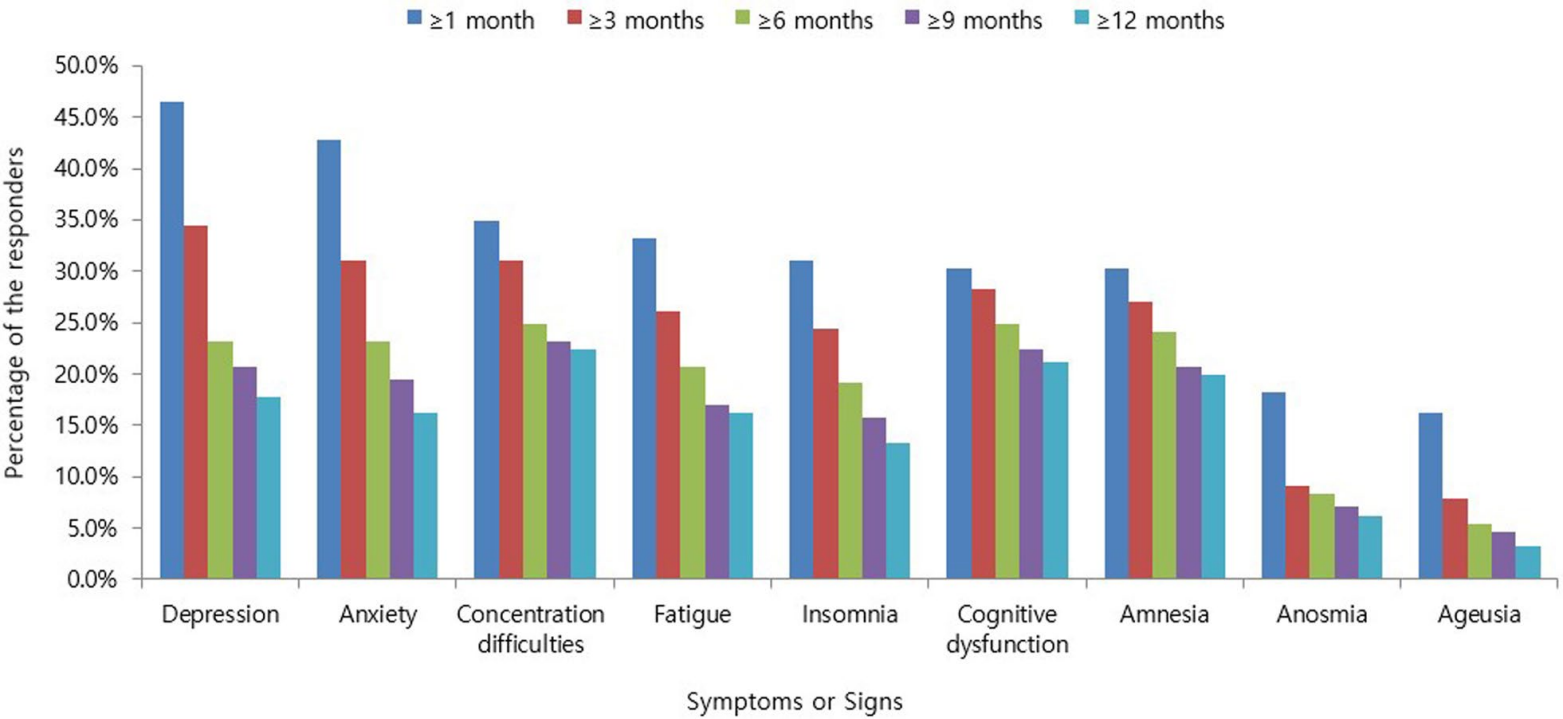
Duration of key persistent symptoms or signs after acute COVID-19 infection

**Fig. 4:**
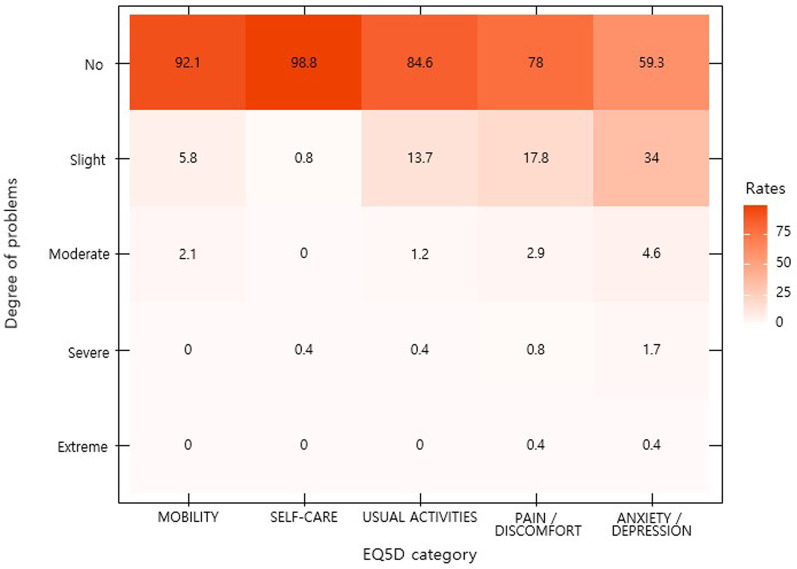
Assessment of quality of life (EQ5D)

**Fig. 5:**
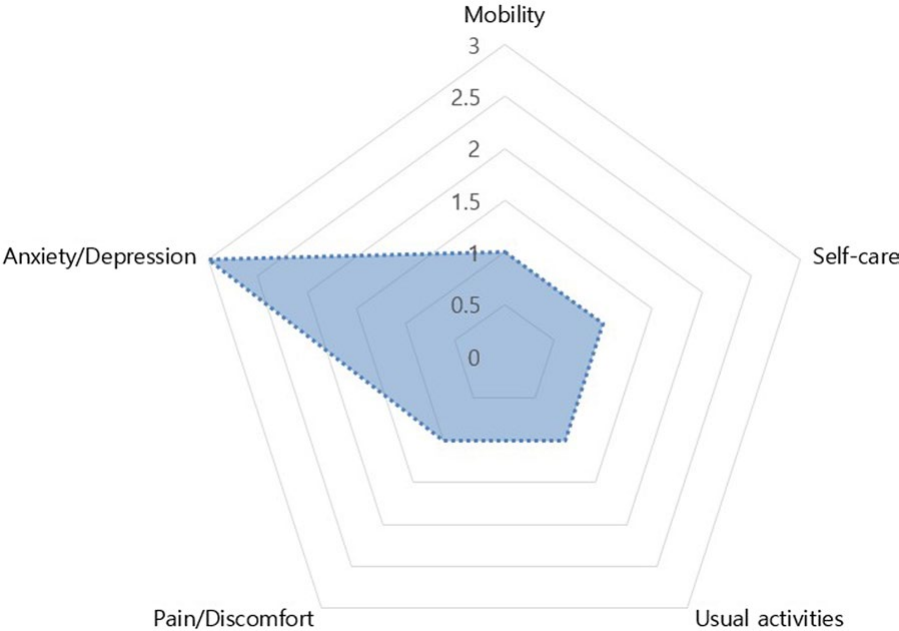
Distribution of EQ-5D median values after 12 months from COVID-19 infection. Each domain of EQ-5D is scored on a 5 point scale: 1, no problem; 2, slight problem, 3. moderate problem; 4, severe problem; and 5, unable to do or extreme problem

The most frequent symptom that lasted up to 12 months was concentration difficulty, which was seen in 54 (22.4%) responders, followed by cognitive dysfunction seen in 51 (21.2%) responders, amnesia in 48 (19.9%) responders, depression in 43 (17.8%) responders, and fatigue and anxiety in 39 (16.2%) responders (Fig. 1). Among the symptoms identified during isolation following acute COVID-19 infection, constitutional symptoms, including fever and myalgia, account for the largest number of cases, with a sharp decrease in the number of respondents complaining over time. However, psychiatric symptoms, such as anxiety, depression, insomnia, social phobia, cognitive dysfunction, and amnesia, continued to increase among the number of respondents after 1 month from the onset of COVID-19-related symptoms or diagnosis. After 1–6 months, 6–12 months, and 12 months from the onset of COVID-19-related symptoms or diagnosis, it was confirmed that the persistent symptoms continued to decrease over time. However, unlike other symptoms, psychiatric symptoms were found to be relatively slow to resolve, lasting more than 12 months (Fig. 2). The nine major symptoms of interest (fatigue, anxiety, depression, insomnia, concentration difficulties, cognitive dysfunction, amnesia, anosmia, and ageusia) improved over time. Among the psychiatric symptoms, the percentage of psychiatric improvement was low, especially up to 12 months after COVID-19 diagnosis, as compared to other psychiatric-related symptoms (Fig. 3). EQ5D test for assessment of quality of life confirmed that 19 (7.9%) responders had mobility problems. In total, 3 (1.2%) responders had problems with self-care, 37 (15.4%) with their daily activities, 53 (22.0%) responders with their pain/discomfort, and 98 (40.7%) responders with their anxiety/depression (Fig. 4). Among the COVID-19-related persistent symptoms, psychiatric problems had a greater impact on quality of life than any other symptoms (Fig. 5).

The nine major symptoms of interest (fatigue, anxiety, depression, insomnia, concentration difficulties, cognitive dysfunction, amnesia, anosmia, and ageusia) were analyzed according to age. Compared with the total number of respondents of each age group, respondents aged 50–59 years most frequent presented with signs of depression, concentration difficulties, cognitive dysfunction, and amnesia symptoms 12 months after the COVID-19-related symptom onset or diagnosis (Additional file 2: Fig. S1). The most frequent symptoms of interest by age group were concentration difficulties at 18–49 years, cognitive dysfunction at 50–59 years, and fatigue at ≥ 60 years (Additional files3: Fig. S2). The most frequent symptoms of interest according to disease severity during acute COVID-19 infection were concentration difficulties in the mild group; anxiety, concentration difficulties, and cognitive dysfunction in the moderate group; and depression, concentration difficulties, and amnesia in the severe group (Additional file 4: Fig. S3). The most frequent symptoms of interest by sex were concentration difficulties and cognitive dysfunction in both women and men, but more frequent in women than in men (Additional file 5: Fig. S4). We divided the study responders into age groups (< 50 and ≥ 50 years), which did not show any difference in the main symptom distribution that lasted up to 12 months after the symptom onset or diagnosis. We found that fatigue, cognitive dysfunction, and amnesia were statistically more frequent among responders aged ≥ 50 years than those aged < 50 years. In particular, cognitive dysfunction showed a difference in the frequency of 14.4% between the two groups (*P* = 0.020) (Additional file 6: Fig. S5). Based on the disease severity at the time of diagnosis, we divided the responders into < moderate and ≥ moderate group to determine the distribution of major symptoms of interest that lasted up to 12 months and found that the frequency of persistent psychiatric symptoms was higher in the ≥ moderate group than in the < moderate group. However, only anxiety symptom was significantly higher in ≥ moderate group than in < moderate group (*P* = 0.022). Anosmia and ageusia continued to have a higher frequency in the < moderate group than in the ≥ moderate groups, but no statistical difference was found (*P* = 0.580 and *P* = 1.000, respectively) (Additional file 7: Fig. S6). In the main sex-specific distribution of symptoms of interest, women were found to have a higher frequency of prolonged psychiatric problems than men; however, intergroup statistically significant difference was only in terms of amnesia (*P* = 0.018) (Additional file 8: Fig. S7).

### Factors associated with the prevalence of persistent symptoms after 12 months of recovery from acute COVID-19 infection

According to the univariate and multivariate logistic regression analysis conducted to identify factors related to prevalence of persistent symptoms after 12 months of recovery from acute COVID-19 infection. For depression (OR 2.34 [1.08–5.68], *P* = 0.043), insomnia (OR 2.84 [1.13–8.65], *P* = 0.040), and amnesia (OR 2.97 [1.36–7.24], *P* = 0.010), the female sex was identified as a relevant factor. For cognitive dysfunction (OR 2.19 [1.11–4.29], *P* = 0.022) and amnesia (OR 2.20 [1.12–4.32], *P* = 0.021), age of ≥ 50 years was identified as a relevant factor (Table 2).

**Table 2:** Univariate and multivariate logistic regression analysis for the factors associated with persistent symptoms or signs after 12 months of recovery from COVID-19

Persistent symptoms or signs	Factors	Univariate OR		Multivariate OR	
		95% CI	*P* value	95% CI	*P* value
Fatigue	≥ Moderate severity	2.33 (0.98–5.24)	0.045	2.02 (0.82–4.72)	0.114
	≥ 50 years old	2.17 (1.06–4.40)	0.031	2.00 (0.94–4.18)	0.066
	Female sex	2.01 (0.91–4.91)	0.099	2.22 (0.99–5.53)	0.066
Anxiety	≥ Moderate severity	2.78 (1.20–6.19)	0.014	2.89 (1.24–6.49)	0.011
	≥ 50 years old	1.10 (0.51–2.28)	0.796	–	–
	Female sex	1.69 (0.79–3.97)	0.198	1.78 (0.82–4.24)	0.163
Depression	≥ Moderate severity	1.13 (0.43–2.67)	0.786	–	–
	≥ 50 years old	1.22 (0.59–2.45)	0.576	–	–
	Female sex	2.34 (1.08–5.68)	0.043	2.34 (1.08–5.68)	0.043
Insomnia	≥ Moderate severity	1.74 (0.65–4.21)	0.242	–	–
	≥ 50 years old	1.33 (0.59–2.88)	0.477	–	–
	Female sex	2.84 (1.13–8.65)	0.040	2.84 (1.13–8.65)	0.040
Concentration difficulty	≥ Moderate severity	2.76 (1.03–4.79)	0.036	2.06 (0.91–4.52)	0.076
	≥ 50 years old	1.79 (0.94–3.38)	0.073	1.63 (0.83–3.17)	0.150
	Female sex	1.86 (0.94–3.93)	0.085	2.01 (1.00–4.31)	0.058
Cognitive dysfunction	≥ Moderate severity	1.81 (0.80–3.92)	0.139	1.51 (0.64–3.41)	0.328
	≥ 50 years old	2.24 (1.17–4.27)	0.014	2.19 (1.11–4.29)	0.022
	Female sex	1.94 (0.96–4.19)	0.077	2.11 (1.03–4.64)	0.051
Amnesia	≥ Moderate severity	1.42 (0.59–3.16)	0.409	–	–
	≥ 50 years old	2.04 (1.05–3.94)	0.033	2.20 (1.12–4.32)	0.021
	Female sex	2.78 (1.29–6.71)	0.014	2.97 (1.36–7.24)	0.010
Anosmia	≥ Moderate severity	0.39 (0.02–2.04)	0.370	–	–
	≥ 50 years old	2.26 (0.76–6.56)	0.129	2.26 (0.76–6.56)	0.129
	Female sex	1.31 (0.43–4.86)	0.651	–	–
Ageusia	≥ Moderate severity	0.81 (0.04–4.74)	0.844	–	–
	≥ 50 years old	4.31 (1.03–21.49)	0.050	4.31 (1.03–21.49)	0.050
	Female sex	0.46 (0.11–1.98)	0.276	–	–

OR odds ratio; *CI* confidence interval

## Discussion

In the present study, we assessed COVID-19-related persistent symptoms in COVID-19 patients after 12 months of recovery from acute COVID-19 infection. In this online survey, 52.7% of the respondents still had at least one persistent symptom. In addition, 5.0% of the total respondents received constant outpatient treatment for COVID-19-related persistent symptoms. Although the COVID-19 related persistent symptoms improved over time, we found that neurocognitive symptoms persisted for 12 months after acute COVID-19 infection. Older age (≥ 50 years), female sex, and disease severity (≥ moderate) were identified as risk factors for neuropsychiatric persistent symptoms.

COVID-19-related persistent symptoms showed broad heterogeneity in terms of frequency and type depending on the time of assessment [14–16]. In a longitudinal prospective study with mild or asymptomatic COVID-19, 34.8% experienced at least one symptom (anosmia, ageusia, fatigue, or shortness of breath) at 7 months [6]. Persistent complications in mild COVID-19 patients decreased over time but were still reported in 28% of the study participants after 12 months of recovery from acute COVID-19 infection [3]. A previous study also showed that 56.9% recovered patients, including the 84.4% patients with moderate or higher disease severity still experienced COVID-19-related persistent symptoms 12 months after acute infection [2]. Patients with higher disease severity had a high rate of persistent symptoms that lasted up to 12 months, but these frequencies may vary depending on the symptoms included in the long-term complication study. Our study involved 194 (80.5%) responders with mild disease severity and revealed that 52.7% patients still complained of COVID-19-related persistent symptoms and the persistency rate was higher than the previous study on mild COVID-19 patients. This frequency difference may be because our study included more diverse types of persistent symptoms.

Of the diverse persistent symptoms, the main symptoms identified in our study were difficulty in concentration, cognitive dysfunction, amnesia, depression, fatigue, and anxiety, which are similar to those reported in previous studies [3, 13]. The possible mechanism of presenting neuropsychological symptoms was explained by a disruption in the blood–brain barrier caused by inflammation, leading to increased permeation of cytokines into the central nervous system. Neuroinflammation from microglial activation and oxidative stress might contribute to delirium in the short term and severe cognitive and functional decline in the long term [17]. Our study demonstrated that neurological symptoms could persist at higher rates for longer periods of time than other symptoms. A study has reported that neurologic manifestations, including cognitive dysfunction induced by COVID-19 infection were the major symptoms in non-hospitalized population [18]. Our study also showed that patients who have recovered from mild COVID-19 can present neurological complications. The strength of our study is that we revealed that mild COVID-19 illness can result in prolonged illness and persistent neurological symptoms, even in young adults and persons with no underlying medical diseases.

We confirmed that the frequencies of neuropsychiatric persistent symptoms were somewhat different even between countries and races. An online survey study conducted in the United Kingdom and United States 7 months after COVID-19 infection showed that 85.1% of respondents experienced brain fog; moreover, cognitive dysfunction and memory impairments were reported in 72.8% of respondents [11]. Our study, in which all participants were of the Asian ethnicity, showed that 47.7% of respondents experienced concentration difficulty, cognitive dysfunction, amnesia, or brain fog after 12 months of recovery from acute COVID-19 infection. It has been confirmed that the number of respondents with long-lasting symptoms in Asian races is relatively lower than in that in other races. This result might be explained by the possibility of coronavirus resistance gene mutation occurring among East Asians as a result of long-term co-evolution of the virus and host [19, 20].

Among the other important neurological symptoms, anosmia has been frequently reported. Anosmia usually shows spontaneous improvement over a period of 2–3 weeks. However, some COVID-19 patients remain anosmic for a longer period [21]. Previous studies have reported that anosmia and ageusia were presented in 27.3% patients with a median age of 72 years after 12 months of recovery from COVID-19 infection [2], whereas 96.1% patients had objectively recovered from the anosmia symptom after 12 months in another study that comprised of women and patients younger than < 50 years [22]. In our study, which was mainly composed of younger patients with a median age of 37 years, anosmia was present in 6.2% of the total respondents. Our result supported the fact that full olfactory recovery is positively associated with younger age [23].

COVID-19 is associated with a substantial and measurable decrease in health-related quality of life [24]. It has been shown that, among young adults aged 18–34 years with no chronic medical conditions, nearly one-third of the respondents reported that they did not return to their usual health after acute COVID-19 infection. Therefore, this suggested that convalescence can be prolonged even in young adults without chronic medical conditions, and thereby potentially lead to prolonged absence from work, studies, or other activities [25]. The findings of our study showed that 15.4% of the responders had problems with their daily activities and 40.7% responders had problems with their anxiety/depression according to the EQ5D instrument for measuring quality of life until 12 months after acute COVID-19 infection.

Patients who had experienced severe COVID-19 had the largest decline in health-related quality of life [26]. Prevention of aggravating COVID-19 with vaccination might be an effective measure to prevent long-term functional decline.

It is unclear why some patients experience long-term symptoms after COVID-19. Potential causes for different outcomes of infection are viral load as well as host-dependent factors, such as genetic susceptibility or induction of anti-inflammatory cells [13]. Increasing age, female sex, disease severity, and body mass index were known as attributes and predictors of persistent COVID-19 infection [27, 28]. A previous study conducted 10 months after the participating patients (median age, 37.8 years) had recovered from an acute COVID-19 infection, similar to our study, showed that older age was associated with greater risk of symptom persistence [29]. Our study demonstrated that factors effecting persistent neuropsychological symptoms after 12 months of recovery from acute COVID-19 infection were associated with age ≥ 50 years and the predominance of women with persistent COVID symptoms, as seen in previous studies [13, 27]. A previous study showed that the presence of post-acute COVID-19 symptoms was more frequently associated with the female sex at 7 months post-COVID-19 infection and that the female sex was associated with depressive symptoms and poor sleep quality, but not with anxiety levels [30]. Our study also showed similar results that persistent symptoms of depression and insomnia were associated with the female sex, but not with anxiety after 12 months of recovery from COVID-19 infection. This study showed that women may be more vulnerable to the impact of sustained neuropsychological persistent symptoms than men. A previous study showed that symptomatic COVID-19 disease with moderate severity, compared with mild severity, was a predictor of persistent COVID-19-like symptoms after discharge [28]. In our study, among the major neurological symptoms, anxiety was identified to be associated with moderate or higher disease severity. Proper targeted interventions can be developed to identify the characteristics of patient groups that are at greater risks of developing long-term persistent neuropsychological symptoms. Early recognition of long-term post-COVID effects and associated risk factors will facilitate diagnosis and multidisciplinary strategies for these patients.

A previous reported the onset of diabetes and worsening of glycemic control in 1.3% and 10.1% of patients, respectively [2]. The role of SARS-CoV-2 in accelerating type-2 diabetes by binding angiotensin-converting enzyme 2receptor on endothelial cells and pericytes of islet microvasculature and pancreatic islet cells, thereby resulting in a local cytokine storm that leads to B-cell dysfunction and apoptosis, has been described [31]. Our study evaluated the onset and worsening of underlying diseases in recovered COVID-19 patients. In this study, which includes more young respondents than previous studies, new onset of diabetes or worsening glucose control was reported in 3.3% of responders. It is suggested that follow-up observations of diabetes may be necessary in recovered COVID-19 patients.

This study has certain limitations. First, since this study was an online survey study, information on persistent symptoms was reported directly by respondents and was not based on an objective evaluation. Second, older patients who found it relatively difficult to participate in the survey were excluded from the study. As a results, this study included most of the young people and those who were able to participate in the online survey by themselves. Therefore, further research of long-term persistent symptoms on patients with severe sequelae or in the elderly population is required. Third, we cannot exclude the contribution of the influence of the long pandemic situation and consecutive psychological impact on the psychological long-term persistent symptoms. Fourth, we were unable to evaluate a control group without COVID-19. Therefore, to accurately evaluate the effects of COVID-19 infection on newly diagnosed diseases, including diabetes, comparative studies with these controls are warranted in the future.

Despite these limitations, our results highlight the need to enhance the preparedness and competence of health-care professionals in the detection and management of the psychological persistent symptoms related to the current COVID-19 pandemic. Moreover, our study showed the importance of future researches on persistent symptoms after COVID-19 infection in vaccinated population, since neurological symptoms can persist until 12 months after recovery from COVID-19 infection, even in patients with mild COVID-19. Whether SARS-CoV-2 infection can promote the development of brain degenerative diseases decades later have not been identified, though it is theoretically possible [32]. Most of the clinical studies about long-term persistent symptoms after COVID-19 infection were conducted in unvaccinated populations. The long-term persistent symptom risk for the fully vaccinated who get infected after vaccination has not been investigated. Future research is needed to clarify whether COVID-19 vaccination can reduce the incidence of long-term persistent symptoms after COVID-19 infection.

## Conclusions

Long-term persistent symptoms were manifested in various forms. We found that neurocognitive symptoms can persist 12 months after recovery from COVID-19, thereby reducing quality of life. Continuous careful observation related to symptom improvement and multidisciplinary integrated research in recovered COVID-19 patients are needed.

## Supplementary Information

**Additional file 1: Table S1.** Sex and age distribution of 241 respondents after 12 months from acute COVID-19 infection

**Additional file 2: Figure S1.** Nine major persistent symptoms of concern according to age group distribution.

**Additional file 3: Figure S2.** Nine major persistent symptoms of concern based on the age groups

**Additional file 4: Figure S3.** Nine major persistent symptoms of concern based on the disease severity groups.

**Additional file 5: Figure S4.** Nine major persistent symptoms of concern based on sex

**Additional file 6: Figure S5.** Nine major persistent symptoms of concern based on < 50 and ≥ 50 years age groups

**Additional file 7: Figure S6.** Nine major persistent symptoms of concern based on the < moderate or ≥ moderate disease severity groups

**Additional file 8: Figure S7.** Nine major persistent symptoms of concern based on the sex

## Data Availability

The datasets used in the present study are available from the corresponding author on reasonable request.

## Abbreviations

COVID-19: Coronavirus disease 2019
SARS-CoV-2: Severe acute respiratory syndrome coronavirus 2
EQ5D: EuroQol-5-dimension
IQR: Interquartile range
OR: Odds ratio
