# Supplementary material for: Post-acute COVID-19 syndrome in patients after 12 months from COVID-19 infection in Korea
Source: BMC Infect Dis. 2022 Jan 27;22:93. doi: 10.1186/s12879-022-07062-6 (PMC8793328; doi:10.1186/s12879-022-07062-6)
Supplement: Supplementary file 1 — Additional file 1: Table S1. Sex and age distribution of 241 respondents after 12 months from acute COVID-19 infection [file 12879_2022_7062_MOESM1_ESM.docx]

**Additional Table S1.** Sex and age distribution of 241 respondents after 12 months from acute COVID-19 infection

| **Characteristics** | **No symptom** | | | | **Presence of symptoms** | | | |
| --- | --- | --- | --- | --- | --- | --- | --- | --- |
| **Sex** | Male | Female | Total | ***P*-value** | Male | Female | Total | ***P*-value** |
|  | (N = 48) | (N = 66) | (N = 114) |  | (N = 29) | (N = 98) | (N = 127) |  |
| **Age (years)** |  |  |  | 0.342 |  |  |  | 0.761 |
| < 50 | 34  (70.8%) | 53 (80.3%) | 87 (76.3%) |  | 18 (62.1%) | 66 (67.3%) | 84 (66.1%) |  |
| ≥ 50 | 14 (29.2%) | 13 (19.7%) | 27 (23.7%) |  | 11 (37.9%) | 32 (32.7%) | 43 (33.9%) |  |
| **Age distribution (years)** |  |  |  | 0.041 |  |  |  | 0.932 |
| 17–29 | 15 (31.2%) | 40 (60.6%) | 55 (48.2%) |  | 10 (34.5%) | 34 (34.7%) | 44 (34.6%) |  |
| 30–39 | 9 (18.8%) | 6  (9.1%) | 15 (13.2%) |  | 4 (13.8%) | 12 (12.2%) | 16 (12.6%) |  |
| 40–49 | 10 (20.8%) | 7 (10.6%) | 17 (14.9%) |  | 4 (13.8%) | 20 (20.4%) | 24 (18.9%) |  |
| 50–59 | 11 (22.9%) | 10 (15.2%) | 21 (18.4%) |  | 7 (24.1%) | 22 (22.4%) | 29 (22.8%) |  |
| 60–70 | 3  (6.2%) | 3  (4.5%) | 6  (5.3%) |  | 4 (13.8%) | 10 (10.2%) | 14 (11.0%) |  |

**Additional figure S1.** Nine major persistent symptoms of concern according to age group distribution

**Additional figure S2**. Nine major persistent symptoms of concern based on the age groups

**Additional figure S3**. Nine major persistent symptoms of concern based on the disease severity groups

**Additional figure S4**. Nine major persistent symptoms of concern based on sex

**Additional figure S5**. Nine major persistent symptoms of concern based on <50 and ≥50 years age groups

**Additional figure S6.** Nine major persistent symptoms of concern based on the <moderate or ≥moderate disease severity groups.

**Additional figure S7.** Nine major persistent symptoms of concern based on the sex.
