# Supplementary figures and images for: Post-acute COVID-19 syndrome in patients after 12 months from COVID-19 infection in Korea
Source: BMC Infect Dis. 2022 Jan 27;22:93. doi: 10.1186/s12879-022-07062-6 (PMC8793328; doi:10.1186/s12879-022-07062-6)

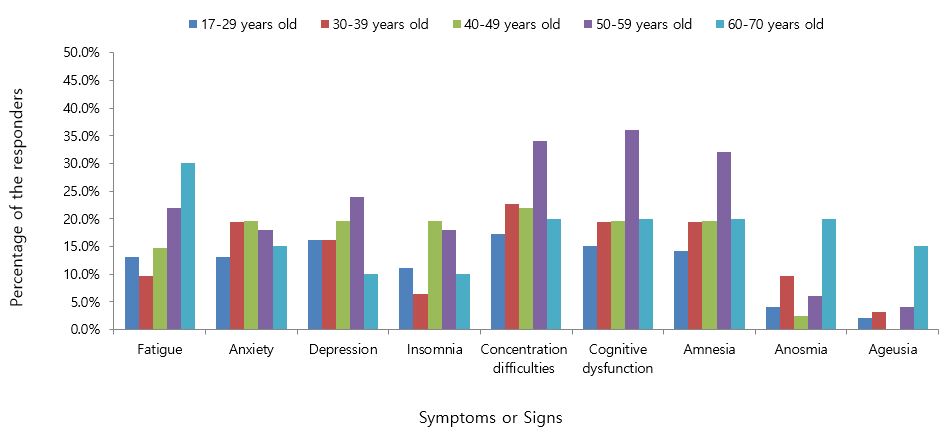

Supplement: Supplementary file 2 — Additional file 2: Figure S1. Nine major persistent symptoms of concern according to age group distribution. [file 12879_2022_7062_MOESM2_ESM.jpg]

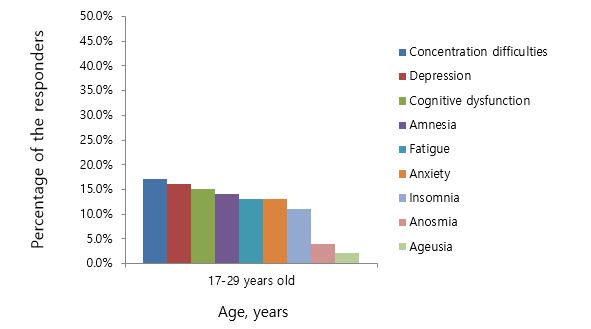

Supplement: Supplementary file 3 — Additional file 3: Figure S2. Nine major persistent symptoms of concern based on the age groups [file 12879_2022_7062_MOESM3_ESM.zip › 12879_2022_7062_MOESM2_ESM/Additional figure S2A.JPG]

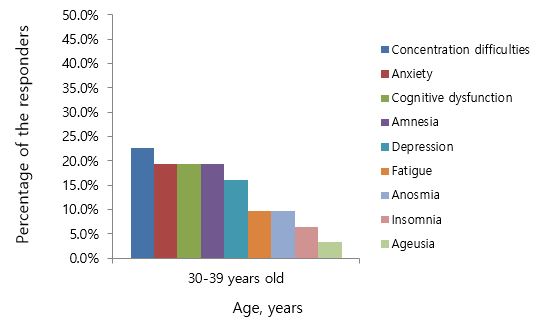

Supplement: Supplementary file 3 — Additional file 3: Figure S2. Nine major persistent symptoms of concern based on the age groups [file 12879_2022_7062_MOESM3_ESM.zip › 12879_2022_7062_MOESM2_ESM/Additional figure S2B.JPG]

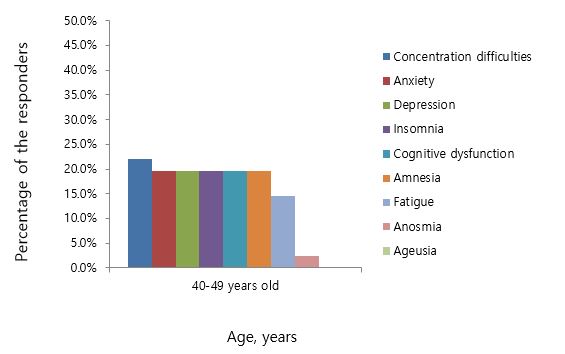

Supplement: Supplementary file 3 — Additional file 3: Figure S2. Nine major persistent symptoms of concern based on the age groups [file 12879_2022_7062_MOESM3_ESM.zip › 12879_2022_7062_MOESM2_ESM/Additional figure S2C.JPG]

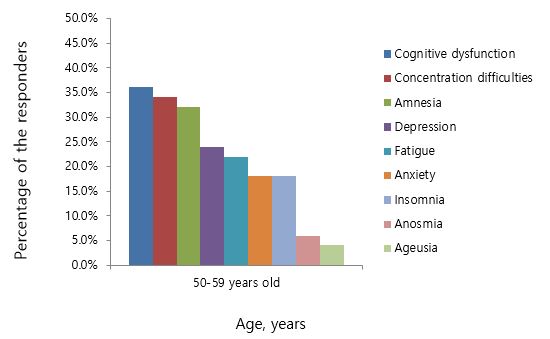

Supplement: Supplementary file 3 — Additional file 3: Figure S2. Nine major persistent symptoms of concern based on the age groups [file 12879_2022_7062_MOESM3_ESM.zip › 12879_2022_7062_MOESM2_ESM/Additional figure S2D.JPG]

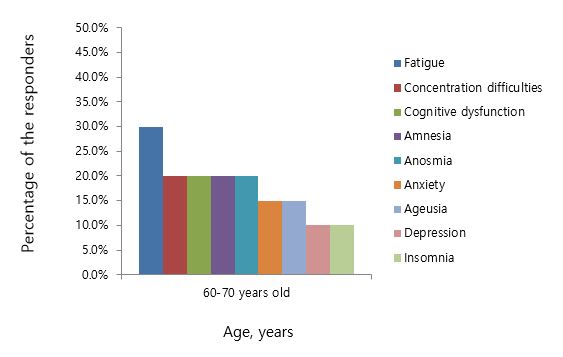

Supplement: Supplementary file 3 — Additional file 3: Figure S2. Nine major persistent symptoms of concern based on the age groups [file 12879_2022_7062_MOESM3_ESM.zip › 12879_2022_7062_MOESM2_ESM/Additional figure S2E.JPG]

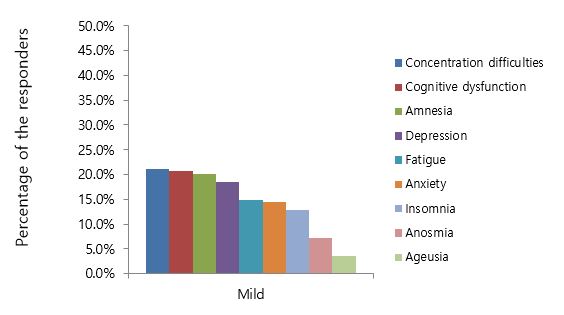

Supplement: Supplementary file 4 — Additional file 4: Figure S3. Nine major persistent symptoms of concern based on the disease severity groups. [file 12879_2022_7062_MOESM4_ESM.zip › 12879_2022_7062_MOESM3_ESM/Additional figure S3A.JPG]

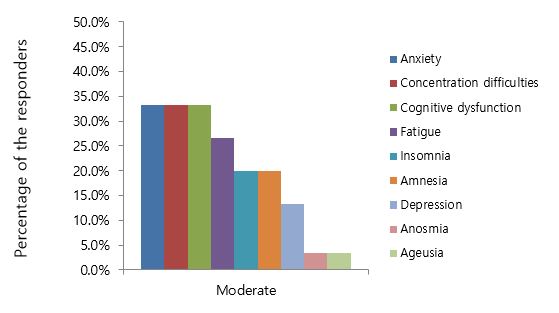

Supplement: Supplementary file 4 — Additional file 4: Figure S3. Nine major persistent symptoms of concern based on the disease severity groups. [file 12879_2022_7062_MOESM4_ESM.zip › 12879_2022_7062_MOESM3_ESM/Additional figure S3B.JPG]

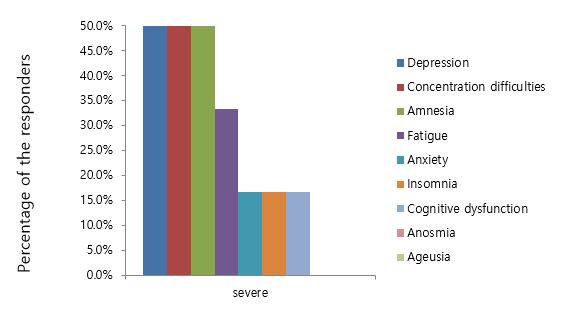

Supplement: Supplementary file 4 — Additional file 4: Figure S3. Nine major persistent symptoms of concern based on the disease severity groups. [file 12879_2022_7062_MOESM4_ESM.zip › 12879_2022_7062_MOESM3_ESM/Additional figure S3C.JPG]

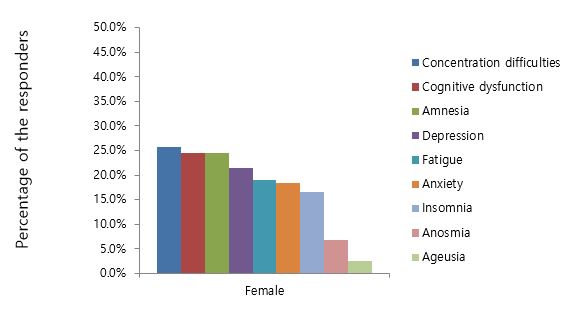

Supplement: Supplementary file 5 — Additional file 5: Figure S4. Nine major persistent symptoms of concern based on sex [file 12879_2022_7062_MOESM5_ESM.zip › 12879_2022_7062_MOESM4_ESM/Additional figure S4A.JPG]

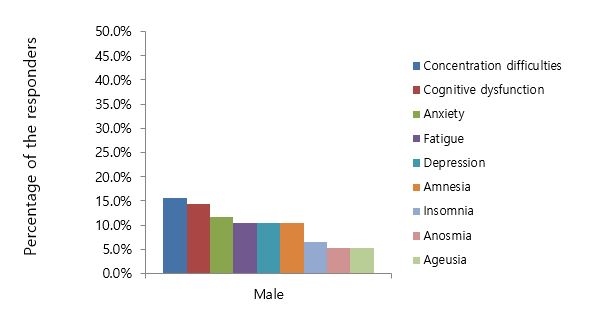

Supplement: Supplementary file 5 — Additional file 5: Figure S4. Nine major persistent symptoms of concern based on sex [file 12879_2022_7062_MOESM5_ESM.zip › 12879_2022_7062_MOESM4_ESM/Additional figure S4B.JPG]

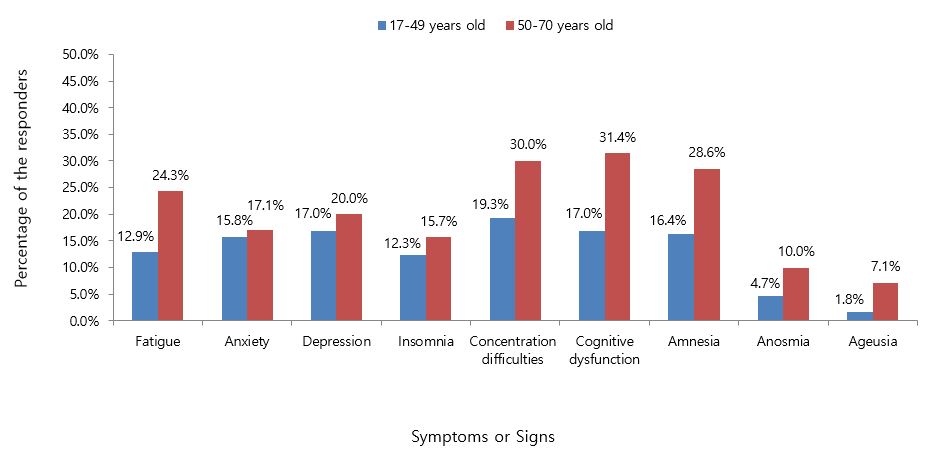

Supplement: Supplementary file 6 — Additional file 6: Figure S5. Nine major persistent symptoms of concern based on < 50 and ≥ 50 years age groups [file 12879_2022_7062_MOESM6_ESM.jpg]

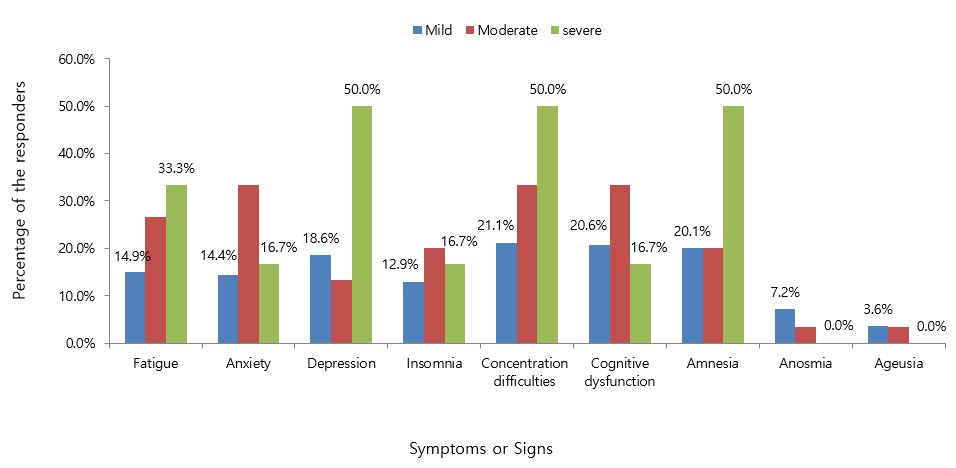

Supplement: Supplementary file 7 — Additional file 7: Figure S6. Nine major persistent symptoms of concern based on the < moderate or ≥ moderate disease severity groups [file 12879_2022_7062_MOESM7_ESM.jpg]

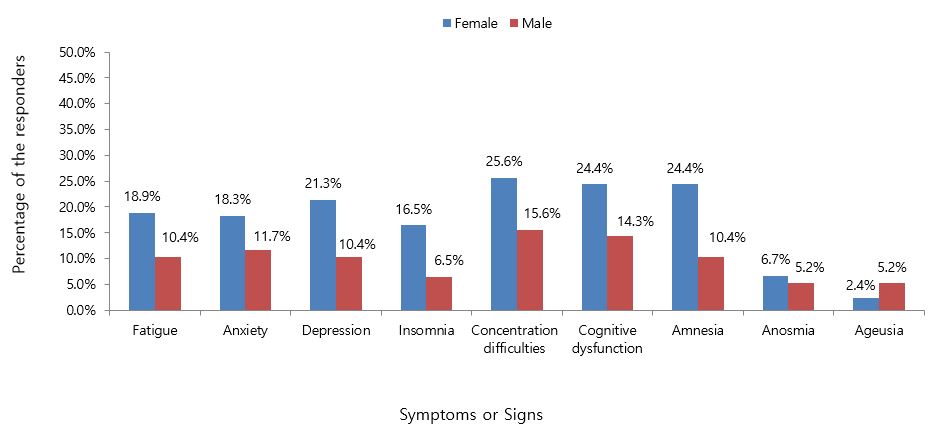

Supplement: Supplementary file 8 — Additional file 8: Figure S7. Nine major persistent symptoms of concern based on the sex [file 12879_2022_7062_MOESM8_ESM.jpg]
